# WHAT CAN WE LEARN FROM THE LINK BETWEEN SLEEP AND GAIT ON THE AGING PROCESS?

**DOI:** 10.1093/geroni/igad104.1619

**Published:** 2023-12-21

**Authors:** Maayan Agmon, Roy Tzemah-Shahar, Tamar Shochat

**Affiliations:** University of Haifa, Haifa, HaZafon, Israel; University of Haifa, Ramat-Gan, HaZafon, Israel; University of Haifa, Haifa, HaZafon, Israel

## Abstract

Falls are a major problem among older adults; and older adults with sleep complaints are at a four-fold risk of falls compared with those without sleep complaints. Recent studies demonstrate comorbidity between gait and sleep, which are two essential, modifiable, and age-sensitive functions. Yet, most studies were conducted on older adults, whereas studies on midlife adults are lacking. Such studies can open a window to better understanding of the aging process. We aim to explore the link between sleep quality and gait among community-dwelling midlife adults. We recruited 103 midlife adults (age 59.3±7). Gait was evaluated with the cognitive Dual-Task (DT) paradigm. Gait speed and variability were assessed with the APDM mobility lab. Sleep quality was evaluated objectively using one-week actigraphy and subjectively using the Pittsburg Sleep Quality Index (PSQI). We found that age was significantly associated with earlier bedtime (p=0.007), lower sleep efficiency (p=0.01) and increased wake after sleep onset (p=0.01). Sex differences were found for objective and subjective sleep duration (actigraphy and PSQI, p=0.002 and p< 0.001, respectively), with women sleeping significantly more than men. No sex differences were found for sleep efficiency. A significant main effect was found for actigraphy-based sleep duration (short/intermediate/long) on gait speed, such that gait speed was slower in long compared to short and intermediate sleepers under DT only (p=0.018). To conclude, a negative association between sleep quality and gait speed under DT was found, showing adverse health outcomes for long versus short and intermediate sleepers in midlife.

